# Simple circuit equivalents for the constant phase element

**DOI:** 10.1371/journal.pone.0248786

**Published:** 2021-03-26

**Authors:** Sverre Holm, Thomas Holm, Ørjan Grøttem Martinsen

**Affiliations:** 1 Department of Physics, University of Oslo, Oslo, Norway; 2 Institute for Energy Technology, Kjeller, Norway; 3 Department of Clinical and Biomedical Engineering, Oslo University Hospital, Oslo, Norway; Information Technology University, PAKISTAN

## Abstract

The constant phase element (CPE) is a capacitive element with a frequency-independent negative phase between current and voltage which interpolates between a capacitor and a resistor. It is used extensively to model the complexity of the physics in e.g. the bioimpedance and electrochemistry fields. There is also a similar element with a positive phase angle, and both the capacitive and inductive CPEs are members of the family of fractional circuit elements or fractance. The physical meaning of the CPE is only partially understood and many consider it an idealized circuit element. The goal here is to provide alternative equivalent circuits, which may give rise to better interpretations of the fractance. Both the capacitive and the inductive CPEs can be interpreted in the time-domain, where the impulse and step responses are temporal power laws. Here we show that the current impulse responses of the capacitive CPE is the same as that of a simple time-varying series RL-circuit where the inductor’s value increases linearly with time. Similarly, the voltage response of the inductive CPE corresponds to that of a simple parallel RC circuit where the capacitor’s value increases linearly with time. We use the Micro-Cap circuit simulation program, which can handle time-varying circuits, for independent verification. The simulation corresponds exactly to the expected response from the proposed equivalents within 0.1% error. The realization with time-varying components correlates with known time-varying properties in applications, and may lead to a better understanding of the link between CPE and applications.

## Introduction

The constant phase element (CPE) is a capacitive impedance with a phase angle in the range 〈−*π*/2, 0〉 which is independent of frequency. It was first introduced by Cole in connection with the electrical impedance of suspensions of spheres [1] and of cell membranes [2]. Jonscher observed that this model is valid for a large range of dielectrics, calling it the “universal” dielectric response. He also connected it to the temporal power law step response of the Curie-von-Schweidler law [3] which was first observed for real-life capacitors more than a century ago. Further Westerlund observed that the response function may be expressed with a non-integer, fractional, derivative [4] and an effective time-varying capacitance with a power-law time variation. The CPE satisfies reciprocity just like ordinary capacitors, i.e. an excitation and the resulting response can be interchanged [5]. In [6] it was also found that the CPE, which fundamentally is linear, could be modeled as a voltage-dependent capacitance which makes it into a nonlinear circuit.

The constant phase element is applied for modeling experimental data from complex systems and here in particular the bioimpedance and electrochemical impedance fields will be highlighted. In the first field, the CPE is a common model for tissue [7, Ch. 9.2.5] along with the related Cole impedance model [8, Ch. 5.8]. Often it is interpreted as a distribution of time constants due to a statistical distribution of cell sizes.

In electrochemistry, CPE behavior is also commonly observed experimentally, and has been interpreted as a statistical distribution of time constants due to for example crystal orientation, surface roughness, and resistance distribution in an oxide layer at the surface [9, Ch. 8], [10, Ch. 13], and [11]. While several physical explanations exist for CPE behavior, such behavior is observed even in idealized experiments on graphite electrodes [12] and equivalent circuit fitting using CPE elements is often used without prior justification. The CPE model is then often used with resistors in series and parallel. That is also the case in tissue bioimpedance [13] as well as in the modeling of supercapacitors [14].

The CPE interpretations in terms of a statistical distribution of cell sizes in the bioimpedance field and the statistical distribution of time constants in electrochemistry will both lead to a band-limited approximation to a CPE as demonstrated in [8, Ch. 7.2]. This multiple relaxation model of the CPE can also be recast into a network model. The simplest example is a lumped cable model where the admittance increases with the square root of frequency for low frequencies, i.e. is described by a half-order derivative. In [8, Ch. 7.4], this is generalized to any order for mechanical models in either a ladder topology or a self-similar tree. Similar topologies may be developed for electrical models also, resembling the topoelectrical circuits of [15].

Here we demonstrate a new interpretation of CPE behavior in terms of simple circuits with time-varying component values. The common capacitive CPE with a negative phase angle will have the same current response to an input voltage impulse as a resistor in series with an inductor that increases linearly with time. An inductive CPE with a positive phase angle will have the same voltage response as a resistor in parallel with a linearly increasing capacitor. The models are inspired by similar ones in linear viscoelasticity [16, 17] where they may model viscosity due to a stick-slip motion between grains in a water-saturated sediment [18].

The paper starts with defining the CPE and connects its frequency and time responses with the fractional derivative description. The proposed linearly time-varying circuits are then analyzed analytically and both exact and approximate solutions are found. The results are confirmed by simulation of the responses of the time-varying circuits in Micro-Cap 12.

## Fractance models

The fractance device is an element with impedance $\tilde{Z}\left(\omega \right)\propto {\left(I\omega \right)}^{\alpha }$ where −1 < *α* < 1 [19, 20]. It is also called a constant phase element, especially if it is capacitive. Here we distinguish between the common capacitive CPE and the less common inductive CPE. Tilde is used to denote a Fourier transform.

### Capacitive constant phase element

The capacitive constant phase element has an impedance given by [4, 6]:

$$\begin{array}{c} \tilde{Z}\left(\omega \right)=\frac{\tilde{u}\left(\omega \right)}{\tilde{i}\left(\omega \right)}=\frac{1}{{\left(\text{j}\omega \right)}^{\alpha }C_{\alpha }},\;0<\alpha \leq 1. \end{array}$$ (1)

where *ω* is angular frequency, $\tilde{u}$ and $\tilde{i}$ are the Fourier transforms of voltage and current respectively, *C*_*α*_ is the CPE parameter in F ⋅ s^*α*−1^ and *α* is the order. The impedance has a negative phase angle of −*απ*/2 which varies from 0 to −*π*/2. The value *α* = 1 gives an ordinary capacitor, *a* = 0 is a resistor, and *a* = 0.5 corresponds to the Warburg element used to model diffusion processes. The CPE model corresponds to an equivalent complex relative permittivity

$$\begin{array}{c} \tilde{\varepsilon }\left(\omega \right)=\frac{C_{\alpha }d}{\varepsilon_{0}A{\left(\text{j}\omega \right)}^{1-\alpha }}, \end{array}$$ (2)

where *d* and *A* are the equivalent plate distance and plate area of the capacitor respectively and *ε*_0_ is the permittivity of vacuum.

The inverse Fourier transform of (1) is a convolution of the input voltage with the current impulse response which here is a temporal power law in time *t*, [8, App. A.3]:

$$\begin{array}{c} i_{imp}\left(t\right)=\frac{C_{\alpha }}{\Gamma (-\alpha )}t^{-\alpha -1},\;t>0,\;0<\alpha <1. \end{array}$$ (3)

The current step response is the integral of the impulse response and is also a power law function:

$$\begin{array}{c} i_{step}\left(t\right)=\frac{C_{\alpha }}{\Gamma (1-\alpha )}t^{-\alpha } \end{array}$$ (4)

This is the Curie-von-Schweidler law [3, 4]. Both responses have an initial singularity which indicates that the CPE model is a simplified and idealized model of a real-life phenomenon. In fact it can be shown that the CPE is an approximation to the more realistic Cole-Cole dielectric model [21] as well as to the Cole-Davidson, and Havriliak-Negami models.

A power law in the frequency domain is one way of defining a fractional derivative, and this broad definition applies to several kinds of operators including the Caputo and Riemann-Liouville ones. The CPE of (1) is therefore equivalent to a fractional capacitor, [4, 8, Ch. 5.8]:

$$\begin{array}{c} i\left(t\right)=C_{\alpha }\frac{{\text{d}}^{\alpha }u(t)}{\text{d}t^{\alpha }}. \end{array}$$ (5)

The fractional operator is a time-invariant operator and in [22] it is shown that the fractional model is linear, has temporal memory, and models slow dynamic electrostatic processes. The associated step response of (4) fits practical capacitors used in electronic circuits with values for the fractional order very close to 1, and examples are given with *α* from 0.9821 to 0.999952 depending on capacitor type. The fit is better than what is achieved with standard equivalent circuits which have a series resistor to account for the resistance of the capacitor’s plates and connectors and/or a resistor in parallel to account for leakage in the insulation. It should be noted that in this work it is the medium model which is fractional, i.e. the complex permittivity of (2) or the equivalent capacitance, (1) and (5). This is in contrast to [23] where there is a fractional dimensional space.

### Inductive constant phase element

An inductive constant phase element where the impedance has a positive phase angle *απ*/2, can be described by

$$\begin{array}{c} \tilde{Z}\left(\omega \right)={\left(\text{j}\omega \right)}^{\alpha }L_{\alpha },\;0<\alpha \leq 1, \end{array}$$ (6)

where *L*_*α*_ is in units of H ⋅ s^*α*−1^ and *α* = 1 gives an ordinary inductance. The voltage is a fractional derivative of the current:

$$\begin{array}{c} u\left(t\right)=L_{\alpha }\frac{{\text{d}}^{\alpha }i(t)}{\text{d}t^{\alpha }}. \end{array}$$ (7)

## Circuit realization of the constant phase element

We model the CPE using time-varying inductors and capacitors. They are examples of linear circuits that do not obey time-invariance that don’t necessarily exist as physical devices although some devices may approximate them. In circuit theory the usual definition is that magnetic flux, Φ(*t*), is the product of inductance and current giving the following current-voltage characteristics:

$$\begin{array}{c} \begin{array}{cc} u(t)= & \frac{\text{d}\Phi (t)}{\text{d}t}=\frac{\text{d}[L(t)\cdot i(t)]}{\text{d}t}\\ = & L\left(t\right)\cdot \frac{\text{d}i(t)}{\text{d}t}+\frac{\text{d}L(t)}{\text{d}t}i\left(t\right), \end{array} \end{array}$$ (8)

where *L*(*t*) is a time-varying inductance. In a time-varying capacitor, current will have a similar relationship with charge. Sometimes a simpler relationship which only includes the first term is assumed instead:

$$\begin{array}{c} u\left(t\right)=L\left(t\right)\cdot \frac{\text{d}i(t)}{\text{d}t}. \end{array}$$ (9)

This ambiguity is reflected in how time-varying inductors and capacitors are implemented in circuit simulators. Micro-Cap from Spectrum Software implements both terms of (8). On the other hand OrCAD PSpice implements the simpler (9), but this is regarded as a problem in [24]. Simulink^®^ from The Mathworks, Inc. gives the user a choice between the two. Here both terms of (8) will initially be used, but it will turn out that since the variation in inductance or capacitive is linear with time in the proposed models, the omission of the last term will not change the final result significantly.

In the field of linear viscoelasticity which has inspired this paper, a simple relationship equivalent to (9) is assumed [25]:

$$\begin{array}{c} \sigma \left(t\right)=\eta \left(t\right)\cdot \frac{\text{d}\varepsilon (t)}{\text{d}t}, \end{array}$$ (10)

where *σ* is stress, *ε* is strain and *η*(*t*) is the apparent time-varying viscosity as used in [16–18]. Therefore, results may not be directly transferable from one field to the other.

### Capacitive constant phase element

**Fig 1:**
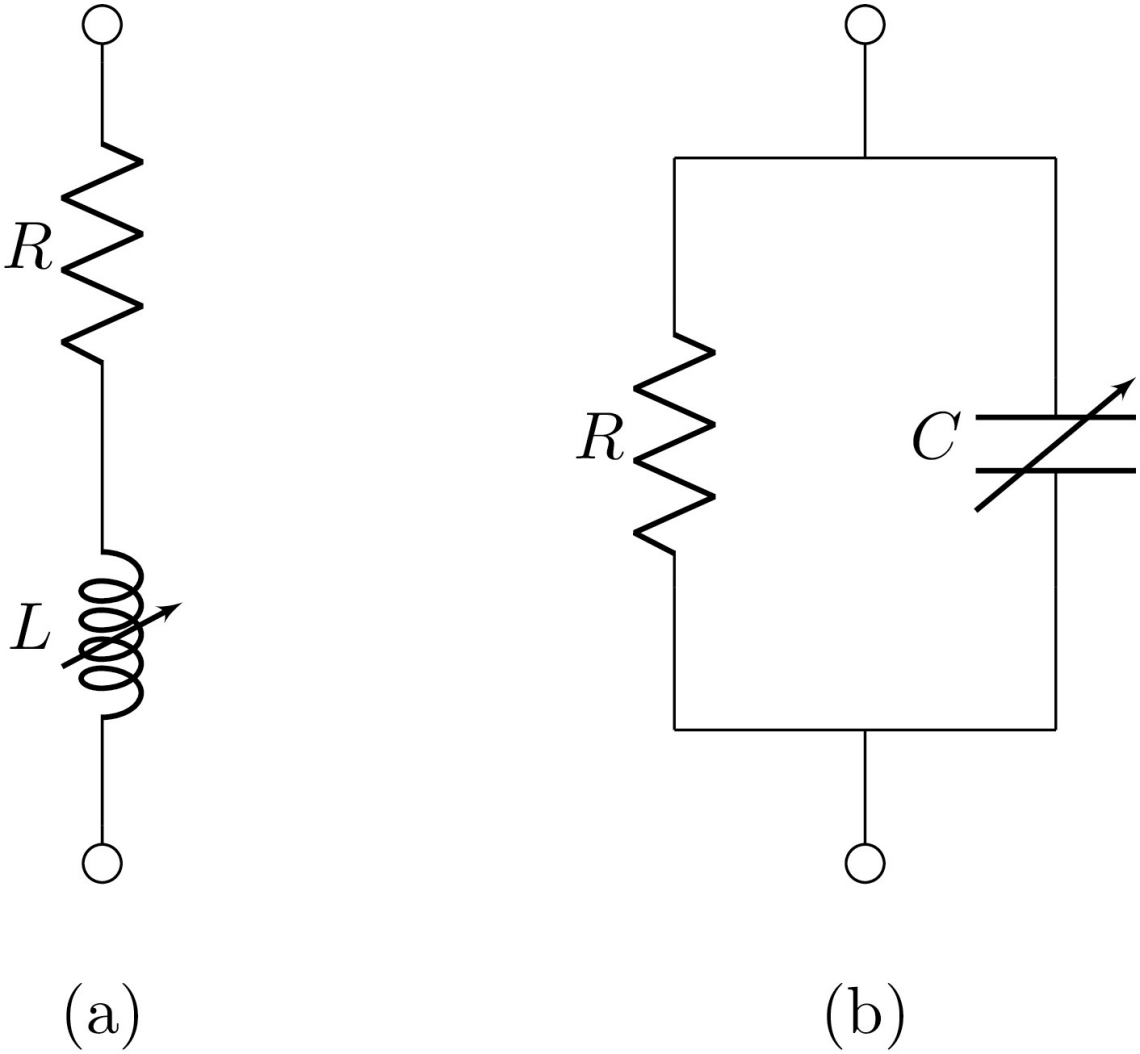
Equivalents to capactive and inductive CPEs. Equivalent circuit for a capacitive Constant Phase Element (a) and an inductive Constant Phase Element (b). Inductance and capacitance respectively increase linearly with time.

The circuit of Fig 1a) where *L*(*t*) = *L*_0_ + *θt*′ is an inductance that increases linearly with time and *θ* has unit H/s = Ω. A second time variable, *t*′ is used here also as is common in time-varying systems. The time of the input impulse, *t*, and the time when the inductance starts changing, *t*′, are in general independent of each other in such a circuit. We will however assume that the variable inductor starts changing at the exact moment when the input impulse is applied so *t*′ = *t*. The voltage-current relationship is then:

$$\begin{array}{c} \begin{array}{cc} u(t)= & Ri+\frac{\text{d}[\left(L_{0}+\theta t^{\prime }\right)i\left(t)\right]}{\text{d}t}{|}_{t^{\prime }=t}\\ = & \left(R+\theta \right)i+(L_{0}+\theta t)\frac{\text{d}i}{\text{d}t} \end{array} \end{array}$$ (11)

It is evident that the only effect of including the second term of (8) in the definition of time-varying inductance is that *θ* contributes to the effective resistance.

By applying a voltage impulse as input at time *t* = 0, the current for positive time after the impulse has occurred, will follow:

$$\begin{array}{c} \frac{\text{d}i/\text{d}t}{i}=-\frac{R+\theta }{L_{0}+\theta t},\;t>0 \end{array}$$ (12)

Following [16, App. 1], integration of (12) gives

$$\begin{array}{c} \ln i=-\frac{R+\theta }{\theta }\{\ln\left(L_{0}+\theta t\right)+\ln K\} \end{array}$$ (13)

where *K* is a constant determined by the initial conditions.

#### Exact CPE

Assume first that the initial value of the inductance, *L*_0_, is zero. Taking the exponential of the previous equation then gives:

$$\begin{array}{c} i\left(t\right)\propto {\left(\theta t\right)}^{-(R+\theta )/\theta },\;t>0. \end{array}$$ (14)

The constant of proportionality cannot in general be found. It can however be determined by considering a practical case related to the following simulation in Micro-Cap. If the input impulse is implemented by exciting one sample of duration *t*_*s*_, the integral of the impulse which is unity, requires a voltage of 1/*t*_*s*_. In that case the initial condition is that *i*(*t*_*s*_) = 1/(*Rt*_*s*_). Thus the final result will be

$$\begin{array}{c} i\left(t\right)=\frac{t_{s}^{\alpha }}{R}t^{-\alpha -1},\;\alpha =\frac{R}{\theta },\;t>0. \end{array}$$ (15)

With positive *R*, *θ*, surprisingly this simple circuit has exactly the same impulse response as the CPE of (3), except for the sign, for all positive values of time. The sign may be changed by allowing for negative resistance, i.e. *R* < 0, possibly also negative inductance, *θ* < 0.

#### Approximate CPE

As it may be hard to imagine a practical time-varying circuit where a component value starts with value zero, we will now let *L*_0_ ≠ 0. Then the solution is:

$$\begin{array}{c} i\left(t\right)=\frac{1}{R}{\left(1+\frac{\theta }{L_{0}}t\right)}^{-(R+\theta )/\theta },t>0. \end{array}$$ (16)

Assuming that *t* ≫ *τ* so that the second term in the parenthesis dominates, this is

$$\begin{array}{c} i\left(t\right)\approx \frac{1}{R}{\left(\frac{t}{\tau }\right)}^{-\alpha -1},\;\tau =\frac{L_{0}}{\theta },\alpha =\frac{R}{\theta },t>0. \end{array}$$ (17)

This result also the same functional form as (3) and therefore this time-varying circuit approximates a CPE. In order to get the sign correct also, one or more parameter values may need to be negative.

### Inductive constant phase element

The circuit of Fig 1b) is parallel to that of Fig 1a) in the sense that voltage and current exchange roles, leading to a current as a function of voltage that resembles (11):

$$\begin{array}{c} i\left(t\right)=\left(\frac{1}{R}+\theta \right)u+(C_{0}+\theta t)\frac{\text{d}u}{\text{d}t} \end{array}$$ (18)

where the time-varying component is now a capacitor and *θ* has unit F/s = S. Therefore, when *C*_0_ = 0 the response will parallel that of (15) and be:

$$\begin{array}{c} u\left(t\right)=R\tau_{s}^{\alpha }t^{-\alpha -1},\;\alpha =\frac{1}{R\theta },\;t>0. \end{array}$$ (19)

Further, it will have a voltage response to an impulse in current which will be of the same form as (17) when *C*_0_ > 0:

$$\begin{array}{c} u\left(t\right)\approx R{\left(\frac{t}{\tau }\right)}^{-\alpha -1},\;\tau =\frac{C_{0}}{\theta },\alpha =\frac{1}{R\theta },t>0. \end{array}$$ (20)

## Verification by simulation

**Fig 2:**
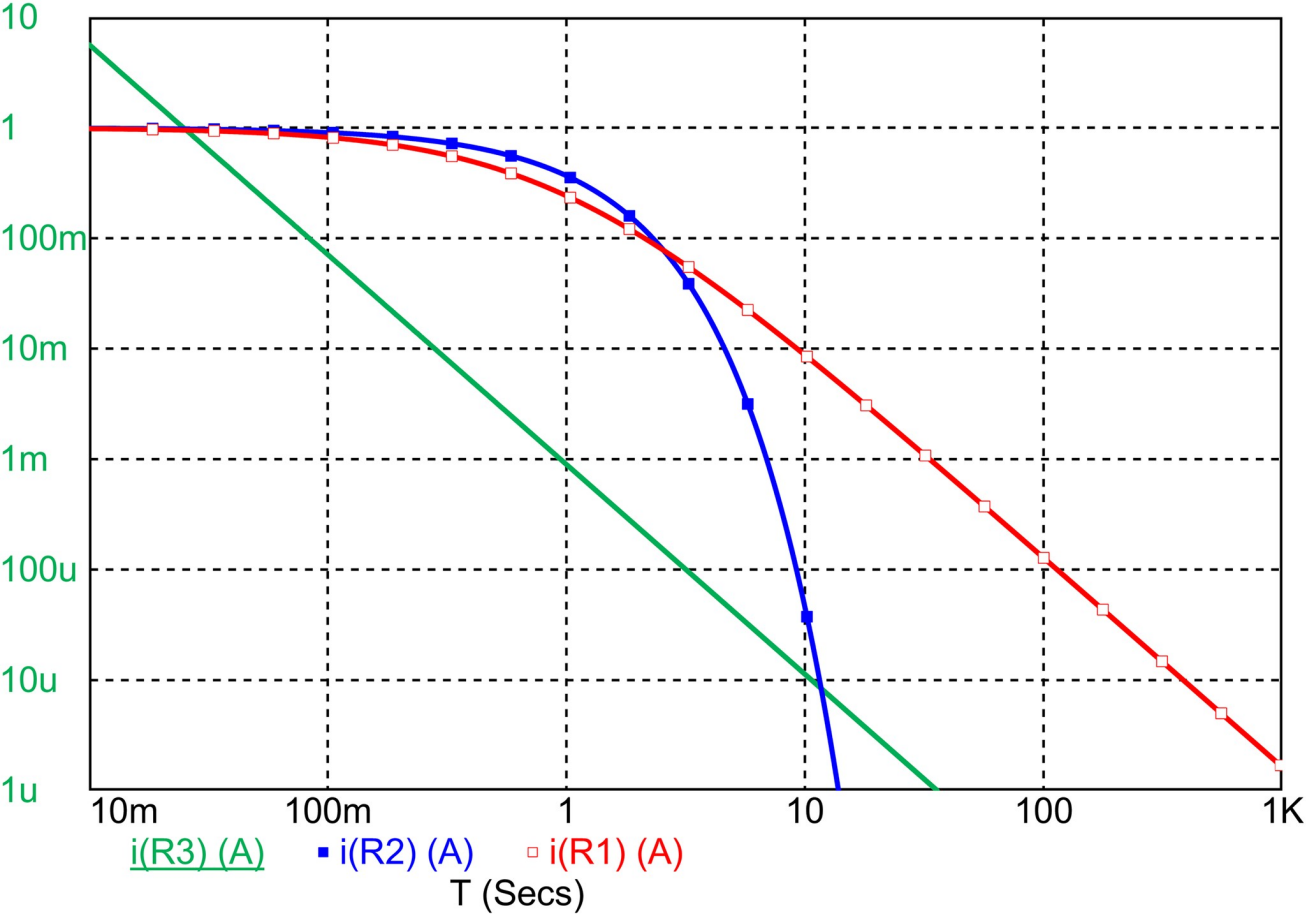
Independent verification. Micro-Cap 12 simulation of current response to a voltage impulse for the circuit of (11) shown in Fig 1a) with *R* = 1, *L* = 1 + *t*/0.9, i.e. *τ* = *α* = 0.9 (red, open squares), *R* = 1, *L* = *t*/0.9, i.e. *α* = 0.9 (green, solid) compared to an ordinary RL-circuit with response e^−*t*/*τ*^/*R*, *τ* = *L*/*R*, *L* = 1, *R* = 1 (blue, filled squares). Pulse length is *t*_*s*_ = 1 ms.

Micro-Cap version 12 [26] is a versatile tool for simulating complex circuits. It is used for the normalized case with positive component values, *R* = 1, *L* = 1 + *t*/0.9, and thus *θ* = *α* = 0.9, and pulse length *t*_*s*_ = 1 ms as shown in Fig 2. The Micro-Cap 12 simulator is now freely available and the circuit diagram and circuit file can be found in the S1 and S2 Files.

It should be remarked that the input to the simulation is the time-varying circuit of Fig 1a) as given by (11). Its agreement with the solutions of (14) and (16) is therefore independent confirmation of their validity. In this figure as well as the two next ones, a comparison is also made with a time-invariant circuit with *R* = 1, *L* = 1 with an exponential response.

**Fig 3:**
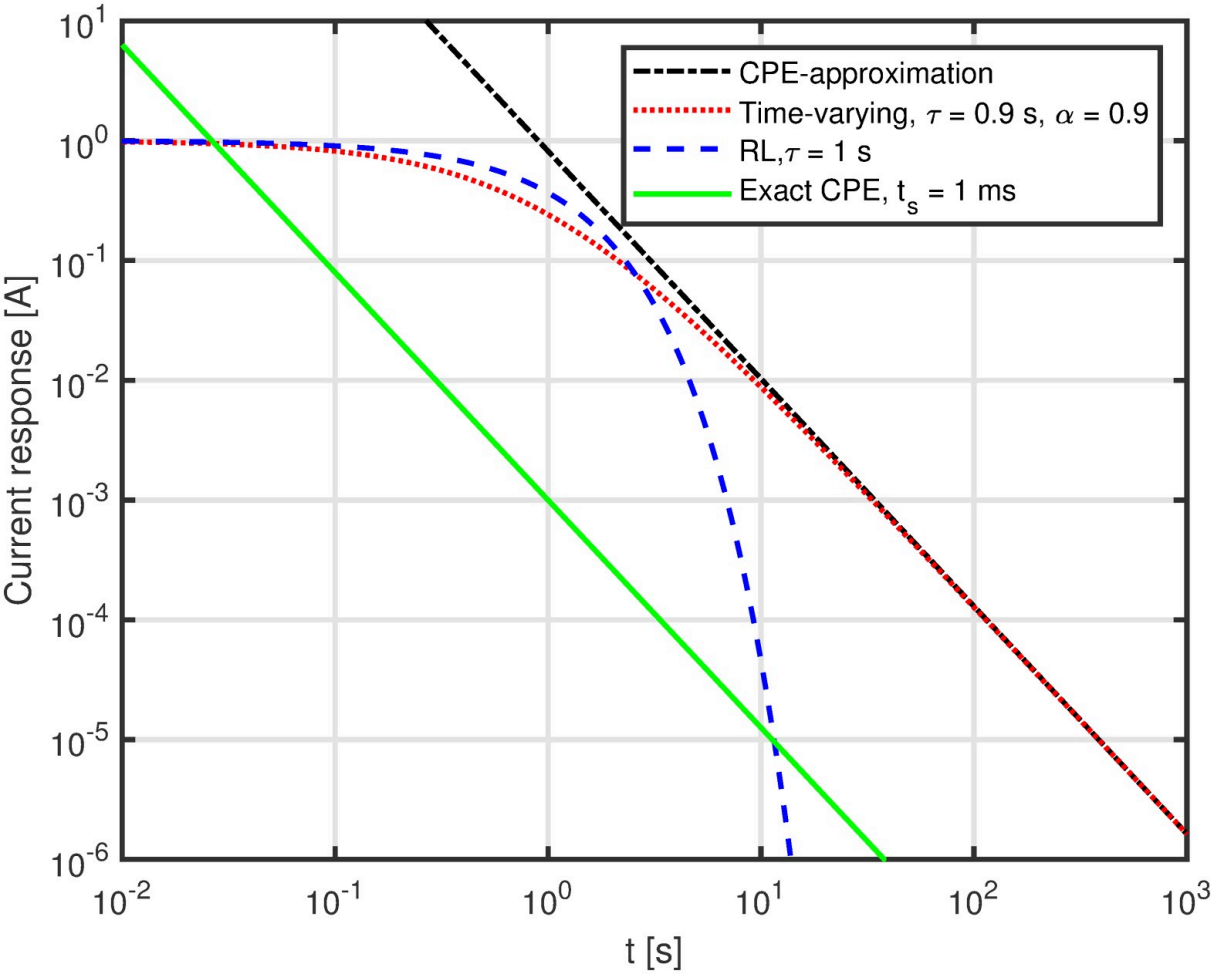
Plot of analytically found response. Current response to a voltage impulse for the circuit of Fig 1a) for *α* = 0.9 computed in Matlab from (15) (green, solid line), (16) (red, dotted line), and (17) (black, dash-dot line) compared to a standard RL-circuit (blue, dashed line).

**Fig 4:**
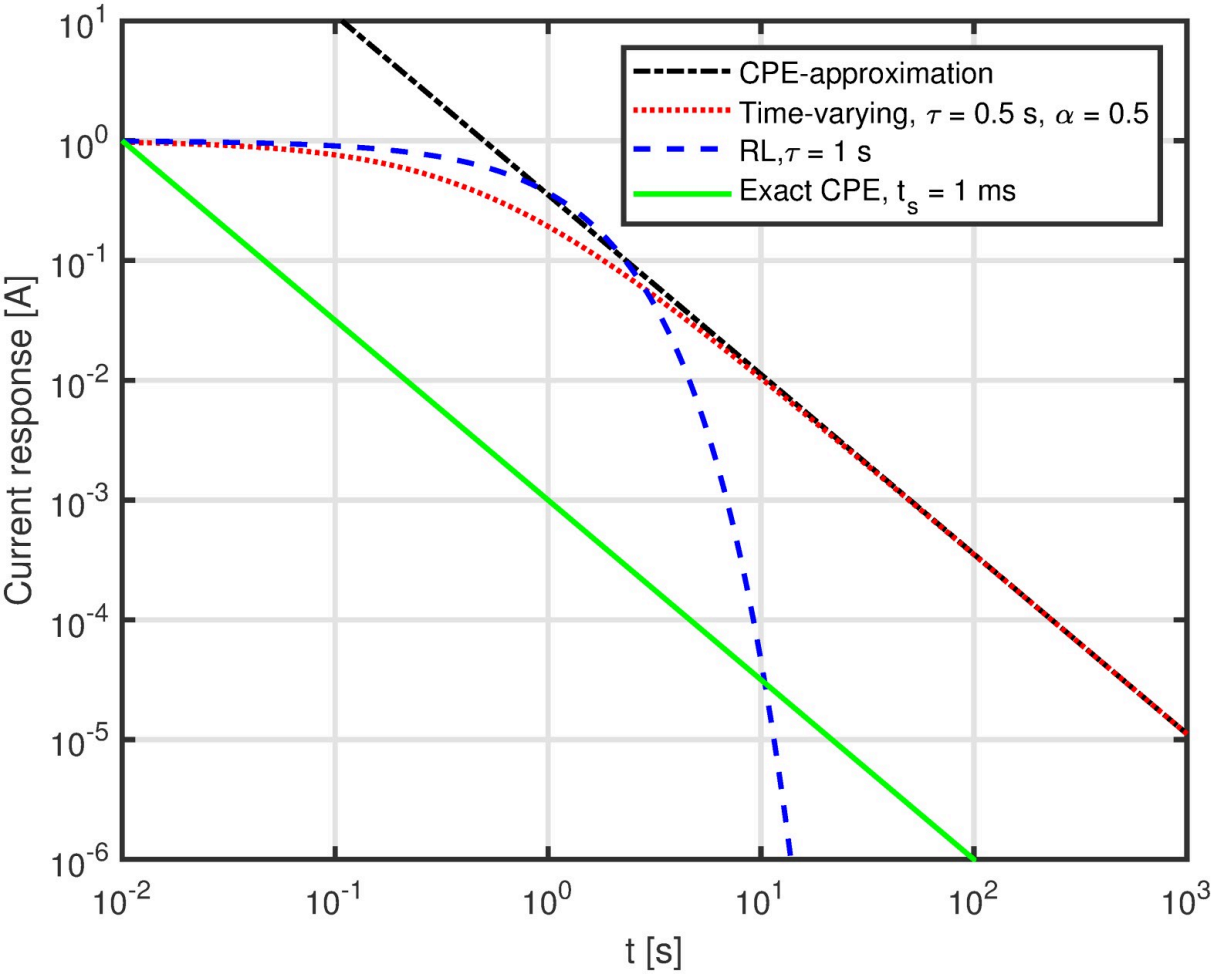
Plot of analytically found response. Current response to a voltage impulse for the circuit of Fig 1a) for *α* = 0.5 (Warburg element) computed in Matlab from (15) (green, solid line), (16) (red, dotted line), and (17) (black, dash-dot line) compared to a standard RL-circuit (blue, dashed line).

Eqs (15), (16), and (17) were then implemented in Matlab and plotted on a logarithmic grid. The case for *α* = 0.9 is shown in Fig 3. The discrepancy between the Matlab computation and the Micro-Cap simulation is maximally in the order of 0.1%. The case for *R* = 1, *L* = 1 + 0.5*t* and thus *θ* = *α* = 0.5 corresponding to a Warburg element, is shown in Fig 4.

The exact expression and the CPE approximation are similar for time above 4-5 seconds when *τ* = 0.9, as shown in Fig 3. In the case of *τ* = 0.5, Fig 4, it happens after a few seconds. This is as expected from the theory as the approximation of (17) is valid when *t* > >*τ*.

## Discussion

Modeling of the capacitive and inductive constant phase elements with time-varying circuits does not critically depend on which definition of the current-voltage relationship for the time-dependent inductor one assumes. The second term of (8) only adds *θ* to R in the results. Thus (14) and (16) have (*R* + *θ*)/*θ* rather than *R*/*θ* in the exponents. The functional form of the final result is therefore independent of the definition, but it will change the exponents by 1.

The results of (15) and (17) demonstrate that the time-varying circuit of Fig 1a) has the same current response to an input voltage impulse as the capacitive CPE. It should be noted that due to the lack of time-invariance in the circuit, this does not imply that the opposite is true, i.e. that the voltage response to an input current impulse is the same as for a CPE. Also, the step response is different, as simple integration of the impulse response does not yield the step response in a time-varying system. These are limitations of the model.

There are several examples of time dependent electrical parameters in biological materials justifying the modeling of the CPE with a time-varying circuit. One candidate is the memristance displayed for example by human skin [27, 28]. The resistance changes as a function of the net amount of charge having passed through the material and when the electrical current is reversed, the resistance will change in the opposite direction. Although the change is charge driven, it will appear as a time dependent resistor when applying a periodic AC signal. Similar mechanisms have been reported for capacitance and inductance, named memcapacitance and meminductance [29] and unpublished results from our group indicate that some biomaterials also have memcapacitive properties. Furthermore, bioimpedance measurements will often contain elements of non-linear properties, typically when using small electrodes where the current density in some volumes may exceed the linear range [30, 31].

Another example of a system that resets itself for every polarity change is the charging of a double-layer in an electrochemical system due to an imposed signal. In each period, the applied signal leads to movements of ions in the solution towards or away from the electrode surface. During charging, ions move towards the electrode, and give electrostatic resistance to subsequent movement of ions. Similarly, during discharge, the ions move away from the electrode and give resistance to the discharge process which varies with time.

These properties open the possibility for finding the equivalent of a steady-state transfer response. One way is to let the second temporal variable *t*′ be reset at every zero-crossing. In that case it will be easier to justify an approximate Fourier relation between the impulse response and the transfer function.

A goal is often to translate the non-ideal CPE element into an equivalent capacitor, notable examples being the Brug equation [32], the oxide layer model [33], and the approaches of [34–36]. The interpretation is often limited to specific systems, where the Brug equation is the most widely applied. While limited to a voltage input pulse here, the presented equivalence circuits may contribute to understanding overall CPE behavior and subsequently establishing more robust methods of interpretation.

The time-varying models presented here may also point to a method for implementing a CPE in a practical circuit. An inductor may be implemented with a gyrator realized with one or more operational amplifiers. The effective inductance will be proportional to a product of a capacitor and a resistor and in particular the latter may be easier to make time-varying than an inductor. This method needs further verification as there are limitations with respect to frequency range in practical gyrators [37]. There may also be limitations in handling of transients.

Finally, it should be noted that the exact solution of (15) where *L*_0_ = 0 is applicable to the viscous model of [16, 18] also. In these papers only the approximate model of (17) is discussed, but by setting the constant part of the time-varying viscosity to zero, *η*_0_ = 0, in Eqs (6) and (8) of [16], an exact solution can be found even for the viscous model.

## Conclusion

We have shown that the capacitive constant phase element (CPE) has exactly the same current impulse response as a resistor in series with a linearly increasing inductance. Likewise the inductive CPE has a voltage impulse response which is similar to that of a resistor in parallel with a linearly increasing capacitance. The similarity is demonstrated under the condition that both temporal variables in the time-varying circuit track each other.

The Micro-Cap 12 circuit simulation program which handles such time-varying circuits, is used for independent confirmation that the impulse responses follow the temporal power law predicted by theory. The realization with time-varying components correlates with known time-varying properties in applications, but this property will need to be explored and established more firmly in future work.

## Supporting information

S1 File Micro-Cap 12 circuit diagram. (PDF)

S2 File Circuit file for Micro-Cap 12. (CIR)
