# Supplementary material for: Simple circuit equivalents for the constant phase element
Source: PLoS One. 2021 Mar 26;16(3):e0248786. doi: 10.1371/journal.pone.0248786 (PMC7997031; doi:10.1371/journal.pone.0248786)
Supplement: S1 File — (PDF) [file pone.0248786.s001.pdf]

S1 Supporting Information for "Simple Circuit Equivalents for the Constant Phase Element"

Sverre Holm, Thomas Holm, Ørjan Grøttem Martinsen

February 28, 2021

Micro-Cap 12 circuit diagram

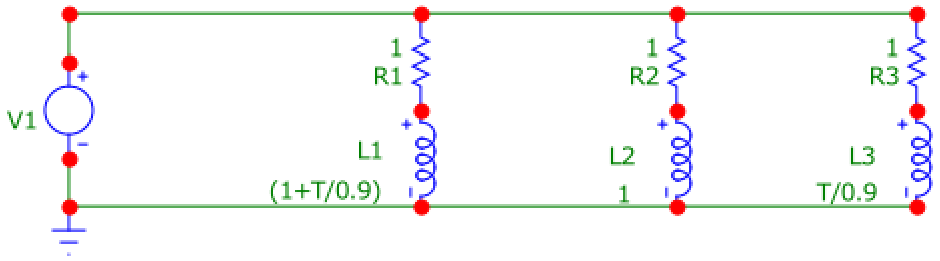

Figure 1: Micro-cap circuit diagram for generating Fig. 3 in main article

The Micro-Cap software has been freely available since version 12.2.0.0, 4 July 2019. See `http://www.spectrum-soft.com/index.shtm`.

See Supporting Information, S2, for the S2-CPE-circuit.cir file that produces this circuit diagram and sets up the simulation.
